# Old Arts and Sciences

**Published:** 1853-04

**Authors:** 


*Old Arts and Sciences.*—We have recently seen a very interesting article under this head in which it is stated that the arts and sciences made considerable progression during the antideluvian age, as did also general knowledge. Rude architecture, the preparation and use of most of our important metals, husbandry, spinning and music were much more generally understood than is supposed, evidenced not only by the writings of profane and sacred historians, but by the recovered wrecks of this world of shade, implying in their adaptation to the use of man, other arts and a considerable advance in science and the mechanic powers. Although our northern friends may claim considerable aptness at guessing, we have no certain knowledge that the surgery of the teeth obtained the least attention until the time of Romulus, 431 years after the Trojan war, when Draco framed his bloody code of laws at Athens, among which was one, requiring the forcing out of the front teeth for certain offences.

